# Sex Disparities in P53 Regulation and Functions: Novel Insights for Personalized Cancer Therapies

**DOI:** 10.3390/cells14050363

**Published:** 2025-02-28

**Authors:** Miriana Cardano, Giacomo Buscemi, Laura Zannini

**Affiliations:** Istituto di Genetica Molecolare Luigi Luca Cavalli-Sforza, Consiglio Nazionale delle Ricerche (IGM-CNR), 20133 Pavia, Italy; miriana.cardano@igm.cnr.it

**Keywords:** p53, sex disparities, cancer

## Abstract

Epidemiological studies have revealed significant sex differences in the incidence of tumors unrelated to reproductive functions, with females demonstrating a lesser risk and a better response to therapy than males. However, the reasons for these disparities are still unknown and cancer therapies are generally sex-unbiased. The tumor-suppressor protein p53 is a transcription factor that can activate the expression of multiple target genes mainly involved in the maintenance of genome stability and tumor prevention. It is encoded by *TP53*, which is the most-frequently mutated gene in human cancers and therefore constitutes an attractive target for therapy. Recently, evidence of sex differences has emerged in both p53 regulations and functions, possibly providing novel opportunities for personalized cancer medicine. Here, we will review and discuss current knowledge about sexual disparities in p53 pathways, their role in tumorigenesis and cancer progression, and their importance in the therapy choice process, finally highlighting the importance of considering sex contribution in both basic research and clinical practice.

## 1. Introduction

In recent years, it has become clear that men are more prone than women to developing non-reproductive tumors, such as lung, liver, brain, bladder, colon and skin cancers and that they also have a worse prognosis and response to therapy [1]. Reasons for these disparities are still unknown even if an important role is surely played by lifestyle, resulting in exposure to different amounts of carcinogens, and by circulating sex hormones that, affecting many different cellular aspects, can also influence tumorigenesis and cancer susceptibility [2].

In recent years, evidence of sex disparities has emerged in many different molecular pathways important for tumor formation and cancer progression that up to now were considered to act equally in male and female cells and that are involved in the regulation of metabolism, senescence, angiogenesis and immunity. One of the most unexpected and intriguing systems of molecular mechanisms showing sex disparities is the DNA damage response (DDR) [3,4].

The DDR is an intricate network of pathways that cells have evolved to prevent chromosomal instability, which can ultimately lead to cancer [5]. The DDR detects DNA lesions and, depending on the severity, either repairs them or promotes premature and permanent cellular senescence or commits the cells to apoptosis (i.e., programmed cell death), thereby restricting their expansion [5].

**Figure 1:**
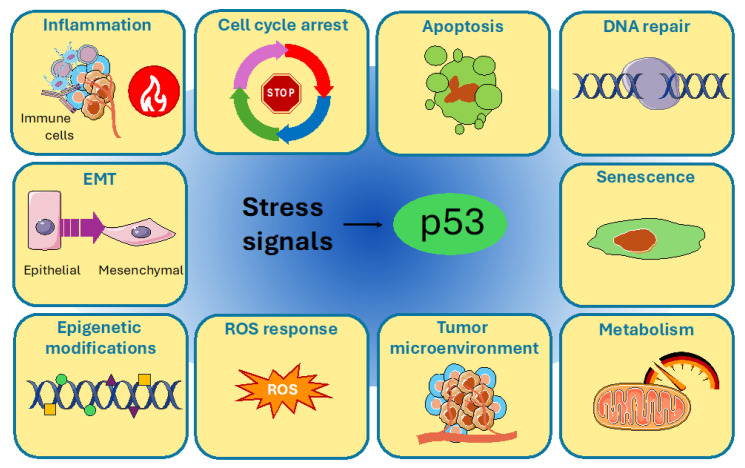
The multiple cellular pathways in which p53 is involved. In response to stress signals, p53 exerts different functions by regulating different mechanisms, which include cell cycle arrest, apoptosis, DNA repair, senescence, cellular metabolism, tumor microenvironment, ROS response, epigenetic modifications, EMT and inflammation. Image was created using pictures from Servier Medical Art, by Servier (http://smart.servier.com).

In human cells, in the presence of particularly complicated or difficult to handle and repair DNA lesions, the DDR is mainly based on two cellular pathways: the ATM-CHK2 and ATR-CHK1 signaling cascades [6]. The ATM-CHK2 pathway mostly responds to double strand breaks (DSBs), while the ATR-CHK1 signaling pathway is activated by replication stress. Upon DNA damage, ataxia telangiectasia mutated (ATM) and ataxia telangiectasia and Rad3 related (ATR) kinases, cooperating with mediator proteins and, respectively, with the transducer kinases checkpoint kinase 2 (CHK2) and checkpoint kinase 1 (CHK1), phosphorylate a multitude of substrates to promote the proper cellular response to lesions, which can be the enhancement of DNA repair capacity, cell cycle arrest, senescence, or apoptosis induction [5]. Since single-strand DNA (ssDNA) stretches are also produced during the first steps of DSBs repair, and DSBs can also result as a secondary effect of replicative stress, cells frequently activate both pathways. A common target of both the ATM-CHK2 and ATR-CHK1 signaling cascades is the tumor suppressor protein p53, a transcription factor mainly involved in the maintenance of genomic stability whose loss or mutation is responsible for tumor susceptibility [5,7,8,9,10]. In response to DNA damage, full activation of p53 is driven by post-translational modifications (PTMs) which direct it to determined promoters to induce the transcription of specific genes, with the purpose of promoting the proper cellular outcomes, generally cell cycle arrest or apoptosis, but also DNA repair and premature senescence, finally creating a barrier against tumorigenesis (Figure 1) [7,8,9,10]. In addition to the response to DNA damage and the maintenance of genome integrity which gained this protein the title of “The guardian of the genome” [11], p53 has been reported to have important roles also in the regulation of metabolism, ROS response, epigenetic modifications, epithelial–mesenchymal transition (EMT), and tumor microenvironment and inflammation (Figure 1) [8].

How these latter stresses activate and regulate p53 remains more elusive. It is in any case clear that p53 mutations can promote different phenotypes in response to different stress sources, depending not only on the type of DNA lesions but also on the involved tissues and on the status of other genes. Importantly, p53 constitutes the most frequently mutated gene of the human genome in cancers; but whether and how its activity is differently regulated in male and female tumors is still an open question.

Here, we review and discuss the recently emerged sex-specific regulations and functions of p53 and their impact in cancer sex disparities and in p53-based therapeutic approaches, to highlight the importance of considering sex in both basic research and clinical practice.

## 2. p53 Protein Structure and Regulation

Initially discovered as a protein interacting with the Large T antigen of the simian virus 40 (SV40), human p53, the product of the *TP53* gene, was, at the beginning, considered an oncoprotein [12]. Subsequently, bi-allelic mutations of *TP53* were found in both human and mouse tumors, finally demonstrating for this protein a tumor-suppressing role [12]. This function was further confirmed by the finding that more than 50% of human cancers share mutations in this gene and that even p53 wild-type patients bear mutations in genes implicated in the p53 pathway [13]. Since then, many efforts have been made with the purpose of elucidating p53 function and regulation, making *TP53* the most studied gene in the world.

**Figure 2:**
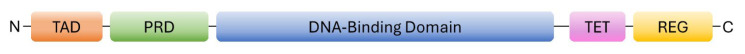
p53 domain structure. Human p53 protein has a transactivation domain (TAD), a proline rich domain (PRD), a DNA-binding domain, a tetramerization domain (TET) and a regulatory domain (REG).

Human p53 is a protein of 393 a.a. characterized by the presence of several functional domains (Figure 2): at the N-terminus, the transactivation domain (a.a. 1–60 is responsible for transcriptional activity, and the proline rich domain (a.a. 61–100) is essential for apoptosis induction; in the central region of the protein (between a.a. 100 and 300) an arginine-rich domain involved in DNA binding is present; at the C-terminus, there are a nuclear localization signal (a.a. 305–322) and a nuclear exclusion domain (a.a. 340–351) regulating p53 subcellular localization, an oligomerization domain (a.a. 326–356) involved in tetramerization, and a negative regulation domain (a.a. 364–393) implicated in activity control [9,13].

**Figure 3:**
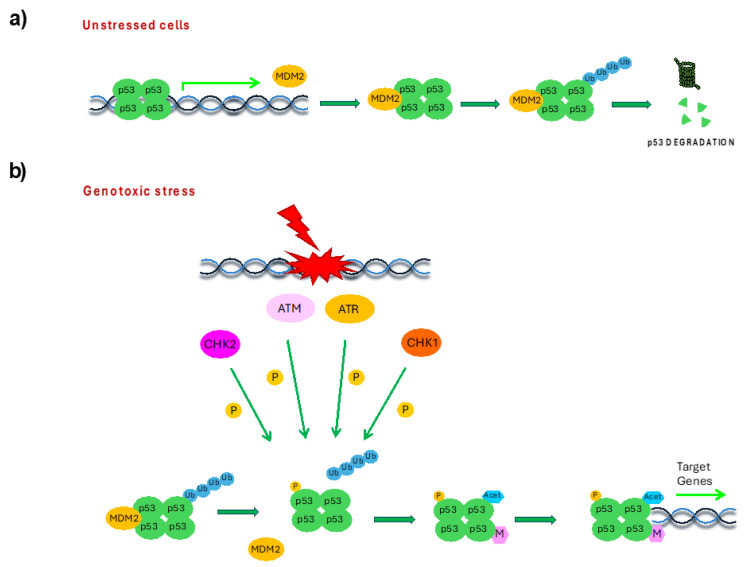
Graphical scheme representing p53 protein level maintenance in unstressed conditions (**a**) and its accumulation and activation in response to genotoxic stress (**b**).

In human cells, p53 is present in a tetrameric state with 20 min half-life. Under physiological conditions, p53 levels are controlled by the ubiquitin–proteasome system, through the involvement of the E3–ubiquitin ligase murine double minute-2 (MDM2), that, by binding the N-terminus and ubiquitinating the C-terminus of p53, drives it to proteasomal degradation. At the same time, p53 promotes the expression of MDM2, establishing an autoregulatory negative feedback loop (Figure 3a) [13]. The importance of the MDM2–p53 complex has been proved by the fact that MDM2-null embryos die by day 6.5 (between 3.5 and 6.5) [14], whereas the lethality is prevented in MDM2-null mice by p53 deletion [15,16]. In response to cellular stress conditions, p53 rapidly accumulates and activates through a mechanism mediated by PTMs. For example, in the presence of DNA damage, p53 phosphorylation by DDR kinases ATR, ATM, CHK1 and CHK2 disrupts MDM2–p53 interaction, preventing p53 ubiquitination and inducing its accumulation [13,17]. Then, the subsequent acetylation and methylation, respectively by acetyltransferases and methyltransferases, of the phosphorylated p53 promotes its full activation and stabilization, by competing with p53 ubiquitination (Figure 3b).

The active form of p53 changes its conformation, exposing the DNA-binding domain and favoring the association with specific promoters, thus inducing transcription of p53-responsive genes involved in cell cycle regulation, DNA repair and apoptosis induction to prevent genome instability and tumorigenesis [13]. Importantly, different types of stress signal, such as defects in ribosome biogenesis, hypoxia, ribonucleotide pool depletion or heat-shock, can produce different combinations of PTMs on p53, also exploiting other protein kinases, acetylases and methylases [8,9,10,13]. These combinations of PTMs direct p53 to specific promoters to induce the appropriate cellular outcome. In this way, the response to different stresses that a cell must cope with are all integrated in a single protein.

## 3. Sex Disparities in p53 Regulation

**Figure 4:**
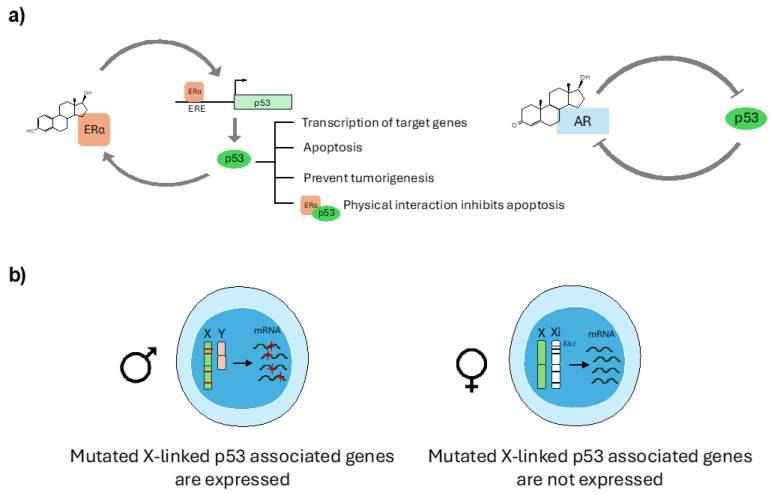
(**a**) Graphical representation of p53–hormones interplay. (**b**) Mutated X-linked mRNA expression in male and female cells. If a gene mutation occurs in the male X chromosome, the mutated gene will be expressed. In female cells, the gene mutations on Xi chromosome will remain unexpressed. Images were created using pictures from Servier Medical Art, by Servier (http://smart.servier.com).

In recent years, evidence of sex disparities has emerged in the regulation of *TP53* gene transcription and p53 protein levels modulation. Indeed, it has been found that sex hormones influence *TP53* gene expression and protein function and that a direct interplay between X-chromosome encoded proteins and p53 does exist (Figure 4a,b).

In addition, p53 PTMs also seem to be differently regulated in males and females. These sex disparities finally impact on p53’s tumor-suppressive role and might contribute to the different cancer predisposition and therapy outcome of males and females.

### 3.1. Sex Hormone Regulation of p53 Expression and Function

Circulating sex hormones, the estrogens and the androgens, have an important role in the regulation of male and female biology and sex steroid receptors are also involved in the DDR. In fact, a reciprocal regulation of DDR genes with androgen (AR) and estrogen receptor α (ERα) has been demonstrated [18]. Regarding p53, its interplay with sex hormones seems to be complex, dependent on the type of tissue or tumor analyzed, and sometimes contradictory.

The role of estrogen has been particularly investigated because of its large use in medicine for contraceptive and cancer therapy purposes. It has been demonstrated that the p53 gene promoter contains 4 ERα responsive elements (ERE) and that estrogen-induced p53 activation is mediated by the binding of the transcription factors CTF-1, NFkB and ERα to p53 promoter [19,20]. On the other side, p53 itself directly promotes ERα expression, establishing a positive feedback loop (Figure 4a) [21]. Accordingly, growing ER+ breast cancer cells in the absence of estradiol (E2) or ERα knock-down (KD) strongly reduces p53 mRNA and protein levels, finally impairing doxorubicin-induced DNA damage-dependent apoptosis [22].

Consistent results were obtained also with mammary epithelial cells obtained by ovariectomized mice or by BALB/c mice with different p53 status and treated with E2 and progesterone (P). In both cases, hormone treatment upregulated p53-dependent signaling and apoptosis upon ionizing radiation exposure, suggesting for these molecules a protective role against breast cancer formation [23,24].

Moreover, in Young Adult Mouse Colon cells (YAMC) it has been demonstrated that estrogen treatment induces the expression of p53 target genes and promotes p53-dependent apoptosis, further confirming the interplay of p53 with hormones in another epithelial tissue [25].

An estrogen-protective role has been observed also in hepatocytes where these molecules promote ERα-dependent p53 expression, finally leading to apoptosis and preventing transformation in hepatocellular carcinoma (HCC) [26,27]. Accordingly, the risk of developing HCC strongly increases in post-menopausal females in comparison with males of the same age, while on the contrary, at younger ages (less than 60 years) men are more susceptible to HCC than women [28], also because testosterone positively regulates cell cycle regulators and reduces p53 expression in hepatocytes [27].

However, contradictory results have also been reported, for example in neural stem cells (NSCs), where UV treatment coupled with administration of sex hormones indicated that estrogen but not testosterone reduces apoptosis in a p53-dependent manner [29]. Other studies suggest that ERα, associating with p53 protein, colocalizes to the promoters of p53 target genes, such as p21^waf1^, PCNA, ATF3, BTG2 and TRAF4, inhibiting their expression and preventing cell cycle progression or reducing doxorubicin-induced apoptosis [30,31,32]. These results therefore suggest that estrogens can activate p53, increasing its expression, but they can also inhibit p53 function through physical interaction, finally leading to different regulation of p53 target genes and different cellular outcomes.

Importantly, the effects of estrogens on p53 protein levels and function seem to be specifically mediated by ERα, since depletion of ERβ demonstrated no significant effects, unless in some colon cancer cell lines where ERβ overexpression increases p53 levels and activity [20,33,34].

Some studies have instead investigated the role of male sex hormones on p53 regulation. Besides its role in HCC, it seems that testosterone may counteract oxidative damage-induced apoptosis in skeletal muscles by inactivating the p53 pathway [35] and that, in prostate cancer, p53 and AR reciprocally inhibit their functions (Figure 4a) [36,37].

In addition, an indirect sex hormone-dependent regulation of p53 has also been reported. Indeed, an MDM2 single-nucleotide polymorphism (SNP) at position 309 (SNP309) substitutes a threonine with a glycine (T > G, SNP309G) in the P2-promoter of MDM2, enhancing its affinity for the transcriptional activator Sp1 [38]. The increased binding of Sp1 induces *MDM2* mRNA transcription and protein accumulation, finally reducing the p53-dependent stress response and increasing the risk for spontaneous tumor development [38]. This SNP is indeed associated with early-onset colorectal cancer, soft tissue sarcomas, diffuse large B-cell lymphoma, non-small cell lung cancer, and invasive ductal breast carcinoma (IDC) [39,40]. Since ER is an Sp1 co-activator, the risk of developing cancer is much more increased in females, especially in the pre-menopausal state [41]. Concordant with an estrogen-dependent female bias, in IDC, SNP309G is associated with ER+ but not ER− breast cancer [40]. Adding to this complexity, MDM2 SNP285C, a second polymorphism found 24 bp upstream of SNP309, alters an ER binding site and disrupts Sp1 affinity. This variant therefore seems to counteract the effects of SNP309 and is associated with a reduced risk in female reproductive tumors [42,43,44].

### 3.2. p53 and X-Chromosome Interplay

A statistical analysis of the 12 most common sporadic tumors in the US population demonstrated that the *TP53* gene is more frequently mutated in male than in female non-reproductive tumors and that different p53 regulatory genes are located on the X-chromosome and mutate more frequently in males than in females [45]. Moreover, a high percentage of mutated X-linked p53-associated genes has been found unexpressed in females, and therefore it is possible that p53 regulatory genes, if mutated, could be specifically inactivated in female cells to protect the p53 signaling cascade (Figure 4b).

One example of X chromosome-dependent regulation of p53 with an impact in therapy outcome is given by the X-linked gene *O-GlcNAc transferase* (OGT). OGT increases p53 stability by adding O-linked N-Acetylglucosamine at Ser149, thus inhibiting p53 binding to MDM2 [46]. In response to chemotherapeutic treatment with 5-azacytidine, the expression of OGT doubles in female fibroblasts, but not in males because of the drug’s ability to block DNA methylation and reduce X-chromosome inactivation (XCI), finally allowing the expression of the inactivated X-linked genes in a sex-specific manner [47].

Similarly, X-linked lysine demethylase 6A (*KDM6A*) has been found to be involved in an epigenetic mechanism that protects females against bladder cancer. In fact, females have an extra copy of *KDM6A* since it escapes XCI, thus promoting p53 tumor suppressor activity by enhancing the expression of p53 gene targets [48].

Importantly, with its 118 miRNAs, the X chromosome encodes for about 10% of the miRNAs in the human genome, therefore representing the highest concentration among all chromosomes [49]. Gene Ontology (GO) analyses indicated that the genes targeted by X-encoded miRNAs are enriched in the p53 pathway, thus indicating another level of X-mediated sex-specific regulation of this cellular mechanism.

In addition, studies with p53-null mice indicated a non-Mendelian rate of male and female progeny. In fact, p53^−/−^ female embryos develop rostral neural tube defects (NTD) that prove fatal, while males have viable caudal NTD [50]. Similar results were obtained also in p53-null rats [51] and with a mouse model expressing the N236S missense mutation of p53 [52]. These sex differences are possibly mediated by p53’s role in XCI [50,53]. In fact, it has now been established that p53 directly promotes the transcription of the lncRNA Xist, which is required for XCI, and, therefore, in p53-null mice Xist levels are reduced, and this causes aberrant XCI and loss of gene dosage compensation that finally results in NTD [53]. Importantly, beyond development, p53 also plays an important role in XCI maintenance in human adult tissues, by ensuring a correct X gene promoter methylation and Xist expression. It has in fact been demonstrated that, in the most aggressive breast cancers, the presence of p53 mutations or low levels of this protein are associated with reduced Xist expression and defects in XCI. This results in the expression of a high number of mutated X-linked genes with important implications for patients’ survival [54].

To balance the dosage of the single active X-chromosome gene expression with that of genes located on autosomes that are in two copies, both male and female cells have evolved strategies to upregulate the active X chromosome. The main regulator of this process is the lysine acetyltransferase KAT8 which, by acetylating histones, specifically promotes the transcription of X-linked genes [55]. However, this protein is only partially accountable for X-gene dosage compensation and abnormal upregulation of X-linked genes have been reported in different diseases, including cancer. Recently, it has been demonstrated that p53, together with its interacting protein ATRX (alpha-thalassemia X-linked syndrome protein), is involved in the repression of abnormal induction of X-linked genes, possibly through restriction of KAT8 expression [56]. Even if the mechanisms underlying these processes have not been fully clarified yet, these findings indicate that p53 is able to regulate X-chromosome gene expression at multiple levels.

### 3.3. Sex Disparities in p53 Post-Translational Modifications

In unstressed conditions, p53 is maintained at low levels by MDM2-dependent protein ubiquitination and proteasome degradation, while after genotoxic stress it is quickly stabilized through PTMs more than transcriptional control [13].

Importantly, in mouse models, sex disparities in p53 PTMs have been recently described, mostly at the level of its upstream regulators in the DDR, ATM and CHK2, revealing findings contrasting with results obtained in human cancers. In C57BL/6 mice, for example, the ATM activity after γ-radiation decreases with age, leading to a reduction of p53 function. However, this decline occurs later in males which survive longer than females [57]. In addition, female mice carrying the CHEK2*1100delC mutation develop tumors more frequently than males [58]. In contrast, germline CHEK2 mutations predispose human females to the development of breast cancer, whereas in lung tissue mechanisms compensating CHEK2 inactivation have evolved. These results therefore indicate sex- and tissue-specific cancer risks that cannot be observed in mice, and suggest that data obtained in mouse models do not always translate to humans.

In addition, as described in the previous section, a sex bias has also been described for p53 O-linked N-Acetylglucosaminylation that increases differently in males and females after chemotherapy, finally interfering with p53 stability [46].

## 4. Sex Disparities in p53 Function

**Figure 5:**
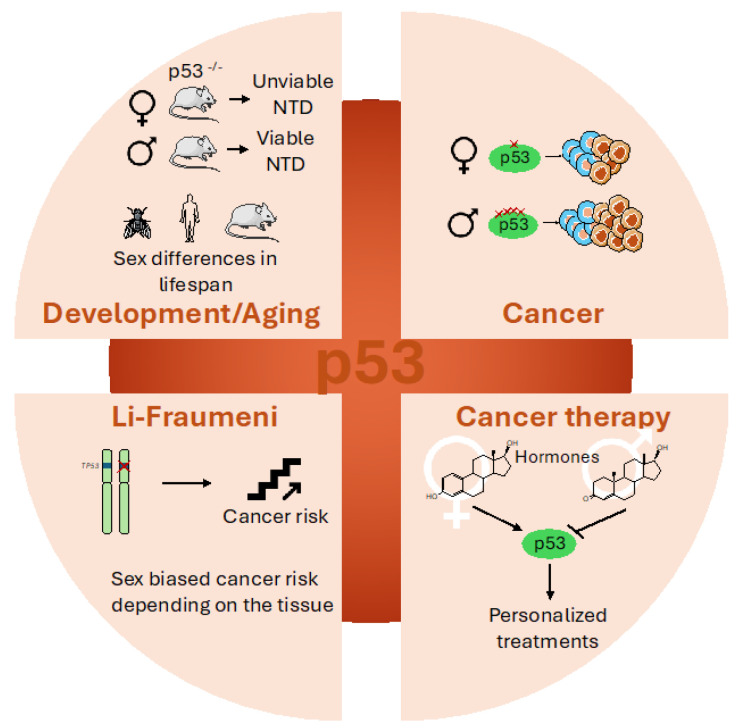
Graphical representation of sex disparities in p53 functions. Sex differences exist in p53’s involvement in development and aging, cancer, Li Fraumeni syndrome and cancer therapy. Image was created using pictures from Servier Medical Art, by Servier (http://smart.servier.com).

In accordance with sex disparities in its regulation, differences in p53 functions have also been found in male and female cells (Figure 5).

However, these disparities are not limited to its tumor-suppressor role, but also impact other pathways necessary for the maintenance of cellular physiology, such as those involved in development and aging, thus underlining the importance of considering sex in all the aspects of p53 function.

### 4.1. Sex Disparities in p53 During Development and Aging

It has been established that sex differences in p53 pathways contribute to sex differences during development. In fact, in addition to the non-Mendelian rate of male and female p53^−/−^ mouse progeny caused by defective Xist expression and NTD occurrence [46,49], it has been demonstrated that p53 plays an important role in the clearance of germ cells that experience DNA damage. These effects are mostly evident during spermatogenesis, when p53 eliminates by necrosis cells with an excessive amount of mutations [59,60]. In females, faulty oocytes are eliminated by the p53 family members p63 and p73 [60,61].

After fertilization, p53 can also influence epigenetic imprinting in a sex-specific manner. Two genes important for fetal development, insulin growth factor 2 (IGF2) and H19, are reciprocally imprinted in mammals, with *IGF2* being methylated and silenced on the maternal allele and *H19* instead being silenced in the paternal allele [62]. Comparison of DNA methylation revealed that the *IGF2* gene is hypermethylated in p53-null female mouse pups, finally reducing *IGF2* expression and promoting birth defects [63].

In addition, p53-null female mice demonstrated defects in embryo implantation independently from the p53 status of the mate or of the offspring. Indeed, loss of p53 results in reduced expression of leukemia inhibitory factor (LIF) gene in the uterus, where it is required for implantation [61,64].

Importantly, p53 is also involved in hematopoiesis. Accordingly, it has been demonstrated that myeloid deletion of heparan sulfate 6-O-endosulfatases (Sulf1 and Sulf2) in mice leads to reduced TGFβ/SMAD2 and enhanced p53/p21^waf1^ signaling specifically in males, finally resulting in sex-specific multilineage abnormalities in bone marrow hematopoiesis [65].

Notably, sex disparities mediated by p53 are evident also during aging. In mice, for example, the population of stem cells in the subventricular zone (SVZ) of the brain decreases with aging, and this loss occurs faster in males than in females. However, p53 deletion eliminates this difference [29], suggesting different functions for p53 in male and female stem cell maintenance during aging.

In addition, always in mice, it has been demonstrated that p53 activity diminishes with aging in a sex-dependent manner [57] and this leads to disparities in longevity, since male C57BL/6 mice have longer lifespans than females. These results, however, contrast with findings in humans where females live longer and develop cancers at a later age than males [66], suggesting that in humans p53 activity may decline in an opposite manner.

In drosophila, p53 seems to have a sex-specific effect on longevity. Indeed, females overexpressing p53 have shorter lifespans than males [67], unless the ectopic expression is restricted to the central nervous system. In this case, p53 overexpression increases female longevity while reducing male lifespan [68].

Curiously, a sex-specific role for p53 in the regulation of chronic pain has also been reported. Four months after nerve injury, male but not female mice show reduced telomere length and p53-mediated cellular senescence in the spinal cord leading to pain maintenance and reduced lifespan. Specifically, p53 positivity in males can be detected in microglial cells which were previously involved in male-specific pain processing. Interestingly, analyses of a UK Biobank also established the relevance of this pathway in humans [69].

### 4.2. Sex Disparities in p53’s Involvement in Cancer

Considering the important role of p53 in the regulation of cell cycle arrest and DNA repair in response to genotoxic stress, it is not surprising that loss of this protein could lead to increased frequency of mutations, copy number variations and aneuploidy, which are all hallmarks of cancer. Accordingly, clonal analysis of tumors identified p53 mutations as an early event in different cancer types, and therefore p53 loss and the subsequent genomic instability have a central role in driving the clonal diversity responsible for tumorigenesis.

Recently, it has emerged that sex disparities in cancer could be due to different incidence of p53 mutations in males and females and to distinct p53 functions between the sexes. In fact, as described above, it has been recently established that, in the 12 most common non-reproductive tumors in the US population, males show an increased frequency of sporadic p53 mutations compared to females, and these genetic changes correlate with poor prognosis and the increased cancer susceptibility of males [45]. However, the reasons for this disparity have not been fully identified yet. Moreover, *TP53* mutations are enriched also in aged men affected by loss of Y chromosome (LOY) in peripheral blood lymphocytes, a condition strongly associated with augmented predisposition to development of malignancies [70].

Besides mutations, SNPs have also been found to affect p53 protein levels and structure, finally influencing its performance and its tumor-protective role in a sex-dependent manner. The most common p53 SNP is the one affecting amino acid 72, which can encode for an Arginine (R) or a Proline (P) with important consequences on p53 structure and performance [71]. In fact, p53 SNP72R promotes mostly apoptosis while p53 SNP72P induces growth arrest and senescence, finally resulting in weaker anti-tumoral activity, but increased longevity [72,73]. However, genome-wide analyses of SNP72 have not found any association with cancer risk [41,74], while a correlation with prognosis has been proposed. Indeed, it has been found that mice with either SNP72P or SNP72R have similar tumor incidence, but mice with SNP72P demonstrated a lower tumor-associated lifespan, compared with SNP72R mice, but an augmented longevity in the case that they do not develop cancer [75]. These studies with mouse models strengthen analyses of the European population that reported a correlation between SNP72P and extended lifespan [76,77]. Unfortunately, these studies did not consider SNP’s effects in males and females independently. Only in one study of German individuals did researchers observe that this difference in longevity was exclusive to females, while no association between the p53 allele and longevity was reported in males [78]. Sex disparities linked to SNP72 in disease were also confirmed in several smaller studies of individual ethnic groups. In a study of Chinese patients with rectal cancer, SNP72P confers extended survival to females but not to males [79]. This SNP is also associated with sex effects in cancer risk in Chinese populations, since males with SNP72P demonstrated an increased risk for Non-Hodgkin lymphoma [80] while females have a higher probability of developing lung adenocarcinoma [81]. In a similar study of the Turkish population, SNP72P is associated with a higher risk of hepatocellular carcinoma only in males [82], while in the Indian population it constitutes a risk factor for males to develop Kangri cancer, a squamous-cell carcinoma of the skin found only in Kashmir [83].

Most recently, it has also emerged that the loss of p53 alternative splicing (AS) isoforms could contribute to tumorigenesis. Indeed, it has been demonstrated that male mice lacking the C-terminus in the p53 AS isoform are more susceptible to Myc-induced B-cell lymphoma than females because of the low expression of the p53 target ACKR4, which is a chemokine receptor important for modulating cellular proliferation and migration [84].

An important role is played by p53 also in protection from glioblastoma (GBM). This male-prevalent tumor is characterized by mutations in the p53 pathway in more than 80% of patients. It has been demonstrated that p53 loss in primary astrocytes lacking the tumor suppressor neurofibromin (NF1) induces sex differences in tumoral transformation, since it promotes cellular proliferation and clonogenic events mostly in male cells [85]. Similar results were obtained also in in vivo experiments where injection of male and female astrocytes lacking NF1 and p53 in the brain of mice of both sexes revealed that male cells promote tumor formation with a high incidence independently of the host’s sex [85]. These findings were confirmed also in a second model of GBM where NF1 and p53 were knocked out in in utero mouse embryos. In this case, all mice developed GBM, but males developed tumors and died faster than females, unless females were depleted of the tumor suppressors RB1, p16 or p21^waf1^, suggesting that female astrocytes have compensatory mechanisms to counteract p53 absence [86]. The majority of p53 mutations in GBM fall inside the DBD. Of these sequence changes, those affecting codons D184, Y205, V216, and V272 are most common in females, while those impacting Y220 and R282 are more frequent in males. These findings suggest that p53 mutations have different gain-of-function effects in males and females that contribute to the selective advantage of these mutations [87].

A hotspot p53 mutation in isocitrate dehydrogenase (IDH) mutant astrocytoma is R273C [88]. Curiously, this genetic change is more frequent in females than in males and, when present, it is associated with a lower number of additional genomic alterations in comparison with alternative *TP53* mutations. However, this relationship is opposite in males [88]. Importantly, compared to other p53 mutations, the Ki67 proliferation index and the expression of other genes associated with cellular proliferation are lower in female IDH mutant astrocytoma with p53 R273C, and these findings are supported also by reduced expression of other genes associated with cellular proliferation. This effect, although present also in males, is more evident in females where the p53 R273C mutation leads to increased expression of p21^waf1^, a well-known inhibitor of cell cycle progression. Conversely, this mutation is associated with reduced progression-free survival and reduced overall survival specifically in males [88].

### 4.3. Sex Disparities in Li-Fraumeni Patients

Patients affected by the familial Li-Fraumeni syndrome (LFS) are characterized by germline mutations in the *TP53* gene that predispose them to different types of tumors, such as breast cancer, soft tissue and bone sarcoma, brain cancer and adrenocortical carcinoma (ACC) [89,90]. LFS patients have a 50% risk of developing tumors before the age of 30 and 90% risk overall during their lifetime. However, a sex bias in cancer development has been described. Indeed, even if the number of mutant p53 carriers is equivalent between males and females, it has been found that females have an increased risk of developing tumors compared to males [91,92]. However, these findings were contradicted by Olivier et al. [93], who observed in LFS patients a male bias in cancer incidence, as found also for sporadic p53 mutations. In this case, it is important to note that studies about cancer and sex in LFS are influenced by the fact that early-onset breast cancer is the most common tumor in women affected by this syndrome. Therefore, the cumulative cancer risk and the annual hazard are strongly influenced by this sex-specific cancer and are therefore higher in females than in males. However, overall cancer risk for tumors unrelated to sex, thus excluding breast and prostate tumors, could be higher in men [94]. Furthermore, focusing on specific cancer types, the risk associated with LFS was found to be different not only when comparing males and females, but also considering sex in association with age [94,95], or with *TP53* mutation type [94]. A female bias was instead confirmed for ACC, which is a rare pediatric cancer strongly associated with p53 mutations [96]. Since this tumor generally occurs in prepubescent children, these data prove that males and females respond differently to p53 mutations depending on the affected tissue and that the effects of p53 mutations, in this case, are not mediated by the action of sex hormones.

### 4.4. Sex-Dependent Effects on p53-Based Cancer Therapy

In consideration of p53’s importance in tumor occurrence, it is not surprising that reactivation of p53’s oncosuppressive function constitutes an important field of study with the purpose of developing personalized cancer therapy. However, sex differences are not consistently considered in both clinical and preclinical trials targeting p53, and separate results for men and women (or their dependence on hormonal status) are not reported. As a consequence, patients’ sex is not yet taken into account during the therapeutic decision-making process and male and female patients are still receiving the same treatments, regardless of their potential to respond to or to be harmed by specific drugs [97].

In particular, the interplay between p53 and sex hormones should be kept in consideration when deciding hormone therapy in tumors with wild-type p53. For example, the anti-estrogenic therapy used to treat breast cancer antagonizes ER function but could also reduce the oncosuppression activity of p53 with negative effects for patients’ treatment. Nonetheless, it has been demonstrated that treatment with 4-hydroxy-tamoxifen, which is an ER modulator, reduces cellular proliferation more efficiently in p53 wild-type than mutant breast cancer cells [98], and fulvestrant, a molecule involved in ER degradation, is able to exert its function independently of p53 status [99].

Importantly, in recent years, different peptides and small molecules have been developed with the purpose of targeting the p53 regulators MDM2 and MDM4, and some of them have entered Phase I clinical trials [100]. However, despite the role of hormones in MDM2 protein level regulation, none of these studies reported evaluations of hormonal status or sex of the enrolled patients. Comparing the efficacy of these molecules in males and females would be very important, since this analysis could highlight the importance of hormones in the regulation of p53 function and could improve the development of personalized cancer therapies.

Recently, it has been established that the CRISPR/Cas9 system is a powerful technology for both gene editing and clinical applications. This system is known to rely on p53 and, since this protein has different regulations and functions in male and female cells, consequently the CRISPR/Cas9 system demonstrated p53-dependent sex-biased effects in cancer cells [101]. Therefore, when deciding to use this system, sex should be taken into consideration, especially during clinical applications.

## 5. Conclusions and Discussion

p53 is the most frequently mutated gene in human cancers and it constitutes a critical tumor suppressor that, activating many different cellular pathways, protects cells from malignant transformation [7,8].

Cancer has recently been acknowledged as a sex-biased disease, since men are more predisposed than women to develop tumors unrelated to reproductive functions and they also have a worse response to therapy [1,4]. Therefore, considering the importance of p53 in tumorigenesis and cancer progression, it is not surprising that this protein could have sex-specific regulation and functions. However, sex-based studies of cancer biology and consequently of p53 are still insufficient and must be improved with the purpose of obtaining novel insights useful for cancer patients’ diagnosis and therapy.

Although p53 is one of the most extensively studied proteins in the world, very little is known about its sex-specific roles. Since p53-targeted therapies constitute promising strategies for cancer treatment [100], understanding the specificity of p53 function and its different interplay with molecules in male and female cells would be of extreme importance in the effort to achieve comparable outcomes for male and female patients. Indeed, up to now, male and female patients are still receiving the same treatments without considering the influence of sex, but generally cancer therapies work better in females than in males [97].

In particular, the relationship of p53 with hormones should be taken into account, especially in consideration of the large use of these molecules for cancer therapy and of their role in p53 expression and function regulation.

We expect that in the next few years, the importance of sex on human health will be fully acknowledged and all pre-clinical and clinical cancer research related to p53 will be appropriately structured to understand sex disparities in both disease occurrence and therapy. These results will help the comprehension of the reasons underlying the greater predisposition of males to cancer and will hopefully contribute to the design of novel strategies for personalized therapies.

## Data Availability

No new data were created or analyzed in this study.
